# Comparison of high or modified low tie of the inferior mesenteric artery in laparoscopic rectal cancer surgery: A meta-analysis

**DOI:** 10.1097/MD.0000000000032065

**Published:** 2022-11-25

**Authors:** Wu Zhong, Chuanyuan Liu, Lei Zhang, Junqiao Zhong, Xianping He, Chuanfa Fang, Hongquan Liu, Laiyang Xia, Zhengyun Zuo, Leichang Zhang

**Affiliations:** a Department of Gastroenterological Surgery, the Affiliated Ganzhou Hospital of Nanchang University, Ganzhou, Jiangxi, China; b Department of Anorectal Surgery, Hospital of Jiangxi University of Traditional Chinese Medicine, Nanchang, Jiangxi, China.

**Keywords:** anastomotic leakage, high tie, inferior mesenteric artery, low tie, postoperative outcomes, rectal cancer

## Abstract

The purpose of this study was to perform a meta-analysis comparing the oncological, intraoperative and safety outcomes in laparoscopic rectal cancer surgery with and without preservation of the left colic artery (LCA).

**Method:**

We searched several databases including PubMed, Web of Science, Cochrane Library, and Embase databases. This meta-analysis included randomized clinical trials, prospective, and retrospective comparative studies regarding high- or modified low-tie ligation of the inferior mesenteric artery in laparoscopic rectal cancer surgery.

**Results:**

Of 641 potentially eligible articles, 16 studies with 3050 participants met the eligibility criteria and were included in the meta-analysis. There was no significant difference in estimated blood loss (WMD −2.63, 95% CI −5.69 to 0.43; *P* = .09), the number of harvested lymph nodes (WMD −0.35, 95% CI −1.60 to 0.20; *P* = .50), the number of apical lymph node yield (WMD −0.19, 95% CI −0.52 to 0.13; *P* = .24), the number of apical lymph node metastasis (OR 0.76, 95% CI 0.40 to 1.45; *P* = .40), rate of conversion to open surgery (OR 0.74, 95% CI 0.50 to 1.09; *P* = .513), rate of urinary dysfunction (OR 1.39, 95% CI 0.71 to 2.74; *P* = .34), rate of recurrence and metastasis (OR 1.10, 95% CI 0.75 to 1.61; *P* = .64), 5-year survival rate (OR 0.89, 95% CI 0.67 to 1.18; *P* = .42). However, this meta-analysis demonstrated a statistically significant difference in operating time (WMD −9.92, 95% CI −15.49 to −5.84; *P* = .0005), rate of diverting stom (OR 1.42, 95% CI 1.06 to 1.92; *P* = .02), rate of anastomotic leakage (OR 2.673, 95% CI 1.91 to 3.62; *P* < .00001), time to first flatus (WMD 0.29, 95% CI 0.11 to 0.48; *P* = .002), time of hospitalization (WMD 0.64, 95% CI 0.14 to 1.15; *P* = .01) between the 2 surgical techniques.

**Coclusion:**

The available evidence suggests that preserving the left colic artery is a safe, effective technique for patients with laparoscopic rectal cancer. nique for patients with laparoscopic rectal cancer.

## 1. Introduction

Colorectal cancer is a major public health problem and the third most commonly diagnosed cancer in men and women worldwide.^[1]^ Radical surgery is considered the treatment offering the best prognosis, but whether the “high tie” (HT) or “low tie” (LT) approach towards the inferior mesenteric artery (IMA) remains controversial. HT involves ligating the artery at its aortic origin, while LT involves ligating it below the origin of the left colic artery (LCA).^[2]^

HT can achieve complete lymphatic clearance, but it may compromise blood supply by sacrificing the left colic artery, thereby increasing the risk of anastomotic leakage.^[3–5]^ HT also increases risk of hypogastric plexus injury, which can impair the urogenital system. In fact, these 2 HT-associated complications can affect patient survival.^[6]^ LT does not increase risk of hypogastric plexus damage, and it partially preserves the left colic artery, although the short length of the preserved artery may hinder tension-free long transplantation in coloanal anastomosis. As a result, LT maintains blood supply, but may increase risk of anastomotic leakage. LT does not achieve complete lymphatic clearance, increasing the possibility of metastasis and recurrence and decreasing survival. Interestingly, previous meta-analyses have reached different conclusions about the incidence of anastomotic leakage with these 2 methods. Fan’s study showed that the incidence of anastomotic leakage in HT group was higher than that in LT group.^[7]^ The results of Shahab and Kong suggested that there was no difference in anastomotic leakage between the 2 surgical methods.^[8,9]^ Si’s results showed that the incidence of anastomotic leakage and urination dysfunction was lower in the LT group than in the HT group.^[10]^ Most studies shown no difference in the number of harvested lymph nodes and 5-year survival between HL and LT.^[7–10]^

Laparoscopic rectal cancer surgery has become more and more common, and its surgical effect is consistent with open surgery is widely accepted. Recently, a modified LT (mLT) technique that dissects the IMA root lymph node and then dissects the IMA below the left colonic artery to achieve D3 lymph node dissection has been widely used clinically, especially in Asian countries.^[11]^ This approach is different from standard LT (procedure without lymph node dissection around the IMA).^[12]^ Some researchers have proposed this approach as an mLT. More lymph nodes can be removed with this procedure, and the patient’s staging and prognosis may be improved.

In previous meta-analyses, open surgery and laparoscopic surgery were combined for analysis, and there was no meta-analyses on the 2 methods of HT and mLT. Further, there have been several randomized controlled trials (RCTs) and retrospective cohort studies published in recent years examining the oncologic outcomes and safety of HL and mLT. Thus, herein we performed a meta-analysis including recently published studies to compare the outcomes of HL and mLT in laparoscopic rectal cancer surgery.

## 2. Methods

### 2.1. Literature search

The databases PubMed, Web of Science, Cochrane Library, and Embase were searched for relevant studies. Literature indexed from 01 January 1980 through 01 June 2021 was searched. The following search terms were used: “rectum,” “colorectal,” “rectal,” “high ligation,” “low ligation,” “intermediate ligation,” “low tie,” “high tie,” “level of arterial ligation,” “left colonic artery preservation,” “selective preservation left colic artery,” “preservation left colic artery,” “left colic artery preserving” and “inferior mesenteric artery.”

### 2.2. Inclusion and exclusion criteria

Studies in any language were eligible for inclusion if they were controlled trials, whether randomized or not, that compared HT and mLT of the inferior mesenteric artery during laparoscopic rectal cancer surgery. The participants of interest included all of the patients undergoing laparoscopic anterior resection for curable rectal cancer. High ligation of IMA, defined as ligation of IMA at its origin from the aorta, and modified low ligation of IMA, defined as ligation of IMA distal to the branch of the left colic artery, radical lymph node excision around the IMA was performed either with high ligation of the IMA at its origin from the aorta or with low ligation below the origin of the LCA branch. Eligible studies were included if their full text was available and if they reported adequate quantitative data about operating time, estimated blood loss, the number of harvested lymph nodes, the number of apical lymph node yield, the number of apical lymph node metastasis, rate of conversion to open surgery, rate of diverting stoma, rate of anastomotic leakage, time to first flatus, time of hospitalization, rate of urinary dysfunction, rate of recurrence and metastasis rates, 5-year survival rate. If studies overlapped in their study populations, only the most recent study was included. Abstracts, letters, expert opinions, systematic reviews and case reports were excluded. Studies were screened initially at the level of title and abstract, then at the level of full text.

### 2.3. Data extraction

Two researchers independently extracted the following data from included studies: first author, publication date, study period, number of patients, country, study design, type of surgery, study inclusion and exclusion criteria, operating time, estimated blood loss, the number of harvested lymph nodes, the number of apical lymph node yield, the number of apical lymph node metastasis, rate of conversion to open surgery, rate of diverting stom, rate of anastomotic leakage, time to first flatus, time of hospitalization, rate of urinary dysfunction, rate of recurrence and metastasis, 5-year survival rate. Disagreements in collected data were resolved by a third researcher.

### 2.4. Assessment of methodological quality of included studies

On the basis of the standards described in the Cochrane Collaboration Handbook, risk of bias of studies contained in this review will be evaluated by all the authors.^[13]^ Disagreements between authors were resolved through discussion. Six criteria items were evaluated for each study, as follows: sequence generation, allocation concealment, blinding, incomplete outcome data addressed, free of selective outcome reporting, and other possible sources of bias.

### 2.5. Statistical analysis

Meta-analysis was performed using Review Manager 5.2 (The Cochrane Collaboration, Oxford, UK). Continuous variables, such as operating time, estimated blood loss, harvested lymph nodes, and apical lymph node yield, time to first flatus and hospitalization were meta-analyzed in terms of weighted mean differences (WMDs) along with 95% confidence intervals (CIs). Reported medians and ranges were converted to means and standard deviations as described.^[14]^ Dichotomous variables such as apical lymph node metastasis, conversion to open surgery, diverting stom, anastomotic leakage rate, urinary dysfunction, recurrence and metastasis, and 5-year survival were meta-analyzed using Petos odds ratios (ORs). Heterogeneity was assessed using Cochran’s Q test and *I*^*2*^ statistics, and *P* < .1 was considered to indicate statistical significance. If heterogeneity was significant, data were meta-analyzed using a random-effects model; otherwise, a fixed-effects model was used. Potential sources of heterogeneity were explored in subgroup analyses. Sensitivity analysis was also performed by removing one study at a time and repeating the meta-analysis. Funnel plots were used to assess potential publication bias.^[15]^

### 2.6. Ethics approval

Given the nature of the present study (i.e. overview of systematic reviews and meta-analyses) and the use of anonymized patient data, requirements for ethics approval were waived.

## 3. Results

### 3.1. Search results

**Figure 1.**
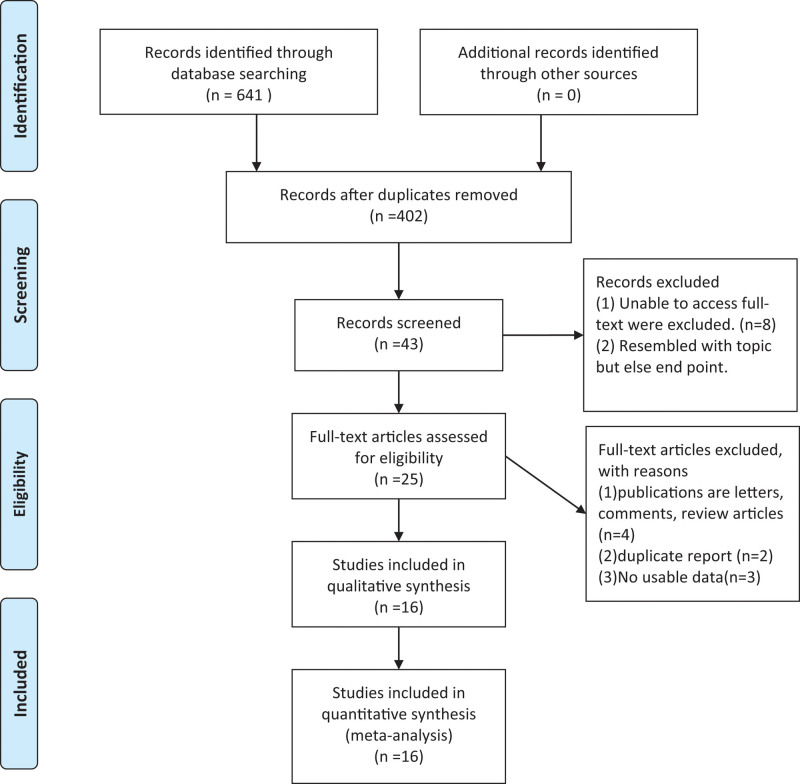
Flowchart of the literature search and selection.

Our original search strategy yielded 641 potential studies (Fig. 1). Excluding duplicates led to 43 studies, of which 8 were excluded because their full text was unavailable, 10 did not report on the required outcomes, 3 were letters or reviews, 1 turned out be a duplicate, and 2 did not report sufficient data. In the end, we included 16 studies in our review.^[16–31]^ These studies, published from 2013 to 2020, included 9 non-randomized controlled studies^[16,18,20,21,26–29,31]^ and 7 RCTs.^[17,19,22–25,30]^ The final set of 16 unique studies contained 3050 patients, of whom 1618 underwent HT and 1432 underwent mLT.

### 3.2. Study characteristics and quality

**Figure 2.**
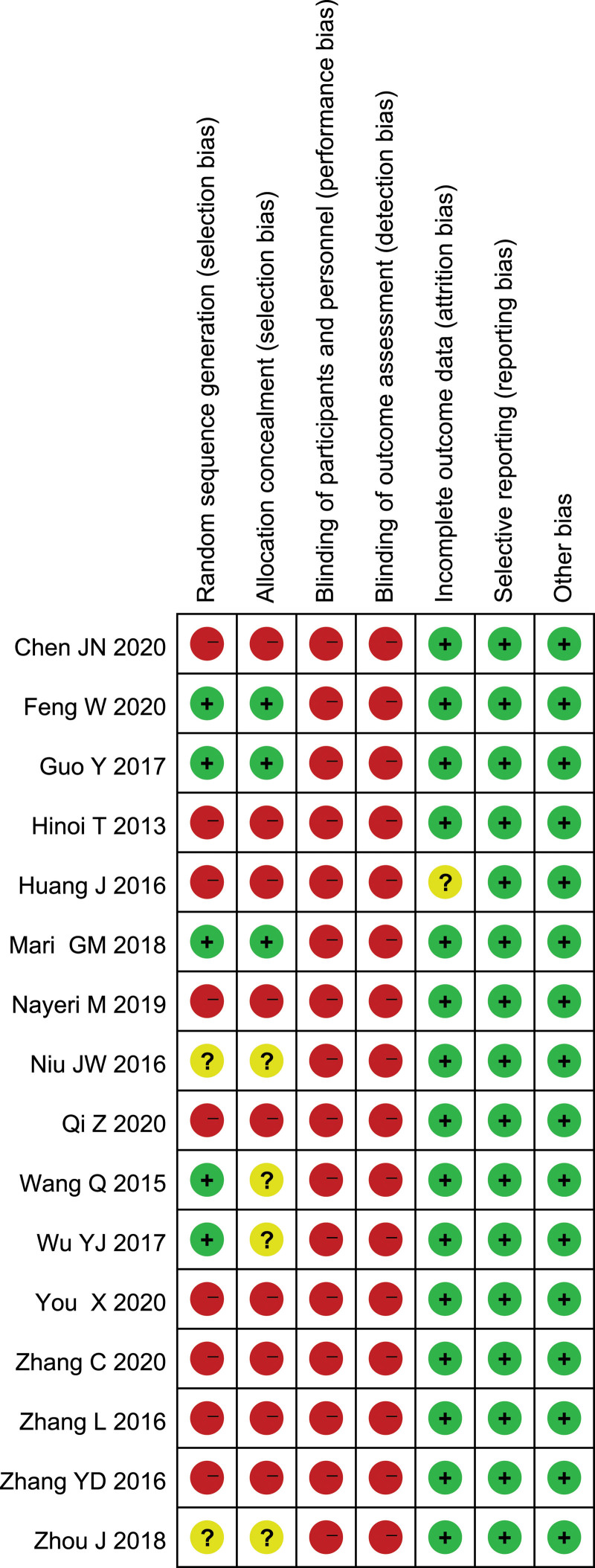
Risk of bias summary.

Patients in 13 studies came from China; 1 study each from Japan, Italy and France. Sample sizes ranged from 57 to 411.The characteristics of the trials included in this meta-analysis are presented in Table 1 The risk of bias summary is presented in Figure 2.

**Table 1:** Characteristics of trials included in the meta-analysis.

Study	Publication year	Country	Study design	N	Treatment (n)		Type of surgery	Adjuvant therapy	Resection of lymph nodes at and around the origin of the inferior mesenteric artery?
					HT	mLT			
Hinoi T^[16]^	2013	Japan	Retro	411	256	155	All LS	No adjuvant therapy	Yes
Wang Q^[17]^	2015	China	RCT	128	63	65	All LS	No adjuvant therapy	Yes
Huang J^[18]^	2016	China	Retro	116	87	29	All LS	All preoperative neoadjuvant chemotherapy	Yes
Niu JW^[19]^	2016	China	RCT	97	45	52	All LS	No adjuvant therapy	Yes
Zhang L^[20]^	2016	China	Retro	103	42	61	All LS	No adjuvant therapy	Yes
Zhang YD^[21]^	2016	China	Retro	216	84	132	All LS	No adjuvant therapy	Yes
Wu YJ^[22]^	2017	China	RCT	96	50	46	All LS	No adjuvant therapy	Yes
Guo Y^[23]^	2017	China	RCT	57	29	28	All LS	No adjuvant therapy	Yes
Mari GM^[24]^	2018	Italy	RCT	214	111	103	All LS	Neo adjuvant CR (HT = 33, LT = 25) Adjuvant therapy (HT = 56, LT = 42)	Yes
Zhou J^[25]^	2018	China	RCT	104	52	52	All LS	No adjuvant therapy	Yes
Nayeri M^[26]^	2019	France	Retro	300	101	199	All LS	Preoperative adjuvant therapy (HT = 25, LT = 27)	Yes
Zhang C^[27]^	2020	China	Retro	205	126	79	All LS	Preoperative chemoradiotherapy (HT = 19, LT = 10)	Yes
Chen JN^[28]^	2020	China	Retro	462	235	227	All LS	Neoadjuvant therapy (HT = 80, LT = 75)	Yes
Qi Z^[29]^	2020	China	Retro	224	116	108	All LS	NR	Yes
Feng W^[30]^	2020	China	RCT	95	47	48	All LS	No adjuvant therapy	Yes
You X^[31]^	2020	China	Retro	322	174	148	All LS	All patients with pathological stage II and above received 6 months of XELOX postoperatively. Those with T4b stage received, in addition, pelvic radiotherapy.	Yes

CR = chemotherapy, HT = high tie, LS = laparoscopic surgery, mLT = modified low tie, NR = not reported, OS = open surgery, RCT = randomized controlled trial, Retro = retrospective.

### 3.3. Operating time

**Figure 3.**
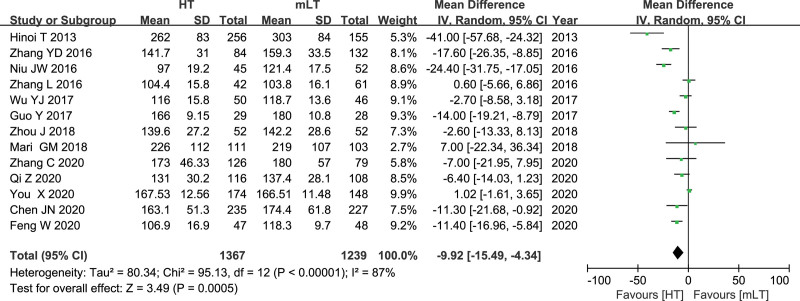
Forest plot of operating time after HT or mLT laparoscopic rectal surgery.

Data on operating time from 13 studies^[16,19–25,27–31]^ involving 2606 patients (1367 HT, 1239 mLT) were meta-analyzed using a random-effects model. There was high heterogeneity among the studies (*I*^2^ = 87%, *P* < .00001). HT was associated with significantly shorter operating time (WMD −9.92, 95% CI −15.49 to −5.84; *P* = .0005; Fig. 3).

### 3.4. Estimated blood loss

**Figure 4.**
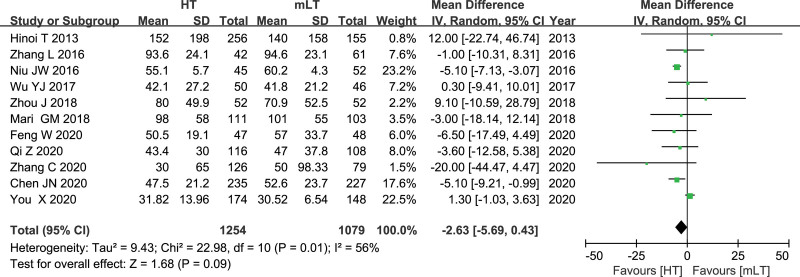
Forest plot of estimated blood loss after HT or mLT laparoscopic rectal surgery.

Data from 11 studies^[16,19,20,22,24,25,27–31]^ involving 2333 patients (1254 HT, 1079 mLT) were meta-analyzed using a random-effects model. There was high heterogeneity among the studies (*I*^2^ = 56%, *P* = .01). HT was associated with similar estimated blood loss as mLT (WMD −2.63, 95% CI −5.69 to 0.43; *P* = .09; Fig. 4).

### 3.5. The number of harvested lymph nodes

**Figure 5.**
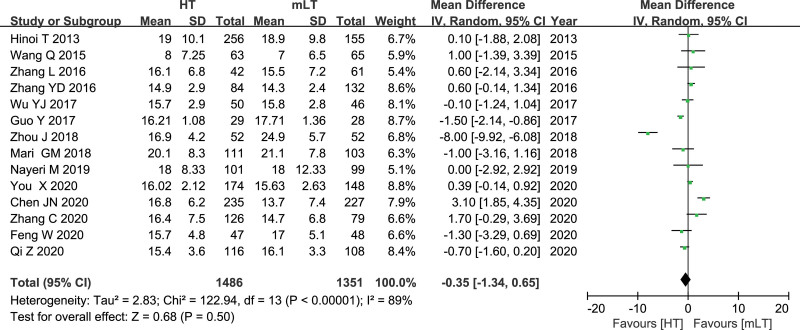
Forest plot of the number of harvested lymph nodes after HT or mLT laparoscopic rectal surgery.

Data from 14 studies^[16,17,20–31]^ involving 2837 patients (1486 HT, 1351 mLT) were meta-analyzed using a random-effects model. There was high heterogeneity among the studies (*I*^2^ = 89%, *P* < .00001). HT was associated with a similar number of harvested lymph nodes as mLT (WMD −0.35, 95% CI −1.60 to 0.20; *P* = .50; Fig. 5).

### 3.6. The number of apical lymph node yield

**Figure 6.**
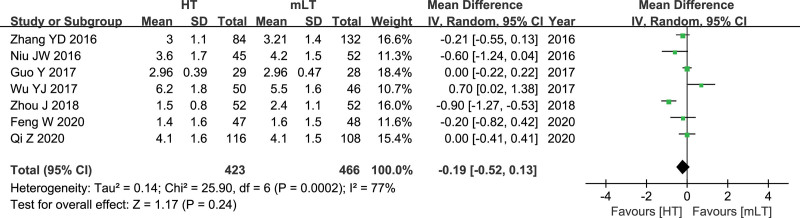
Forest plot of the number of apical lymph node yield after HT or mLT laparoscopic rectal surgery.

Data from 7 studies^[19,21–23,25,29,30]^ involving 889 patients (423 HT, 466 mLT) were meta-analyzed using a random-effects model. There was high heterogeneity among the studies (*I*^2^ = 77%, *P* = .0002). HT was associated with similar apical lymph node yield as mLT (WMD −0.19, 95% CI −0.52 to 0.13; *P* = .24; Fig. 6).

### 3.7. The number of apical lymph node metastasis

**Figure 7.**
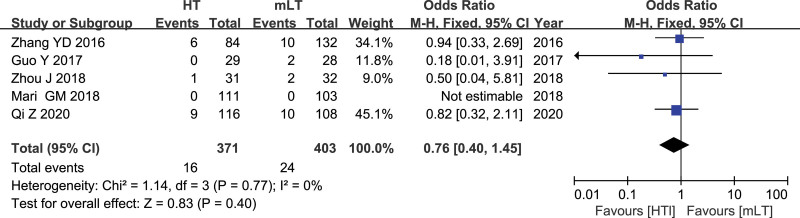
Forest plot of the number of apical lymph node metastasis after HT or mLT laparoscopic rectal surgery.

Data from 5 studies^[21,23–25,29]^ involving 774 patients (371 HT, 403 mLT) were meta-analyzed using a fixed-effects model. There was low heterogeneity among the studies (*I*^2^ = 0%, *P* = .77). HT was associated with similar number of apical lymph node metastasis as mLT (OR 0.76, 95% CI 0.40 to 1.45; *P* = .40; Fig. 7).

### 3.8. Rate of conversion to open surgery

**Figure 8.**
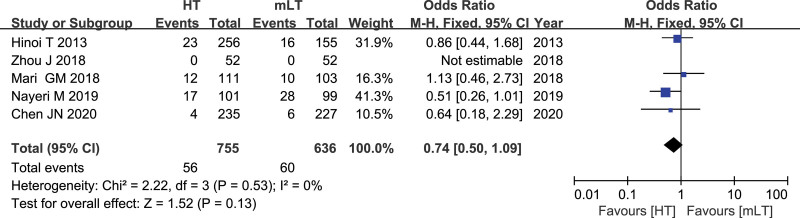
Forest plot of the rate of conversion to open surgery after HT or mLT laparoscopic rectal surgery.

Data from 5 studies^[16,24–26,28]^ involving 1391 patients (755 HT, 636 mLT) were meta-analyzed using a fixed-effects model. There was low heterogeneity among the studies (*I*^2^ = 0%, *P* = .53). HT was associated with similar rate of conversion to open surgery as mLT (OR 0.74, 95% CI 0.50 to 1.09; *P* = .13; Fig. 8)

### 3.9. Rate of diverting stoma

**Figure 9.**
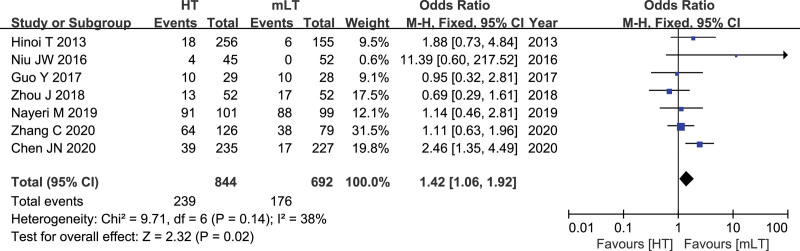
Forest plot of the rate of diverting stoma after HT or mLT laparoscopic rectal surgery.

Data from 7 studies^[16,19,23,25–28]^ involving 1536 patients (844 HT, 692 mLT) were meta-analyzed using a fixed-effects model. There was moderate heterogeneity among the studies (*I*^2^ = 38%, *P* = .14). HT was associated with a significantly higher rate of Rate of diverting stoma as mLTT (OR 1.42, 95% CI 1.06 to 1.92; *P* = .02; Fig. 9).

### 3.10. Rate of anastomotic leakage

**Figure 10.**
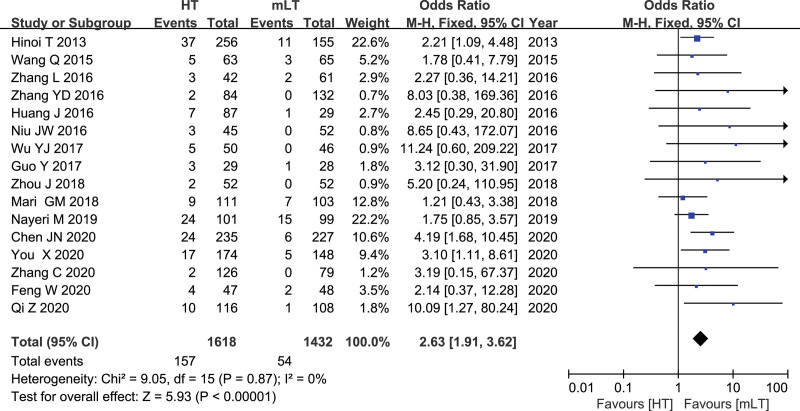
Forest plot of the rate of anastomotic leakage after HT or mLT laparoscopic rectal surgery.

Data from 16 studies^[16–31]^ involving 3050 patients (1618 HT, 1432 mLT) were meta-analyzed using a fixed-effects model. There was low heterogeneity among the studies (*I*^2 ^= 0%, *P *= .87). HT was associated with a significantly higher rate of anastomotic leakage (OR 2.673, 95% CI 1.91 to 3.62; *P* < .00001; Fig. 10).

### 3.11. Time to first flatus

**Figure 11.**
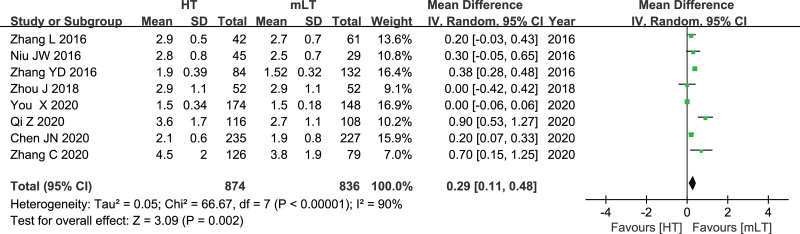
Forest plot of time to first flatus after HT or mLT laparoscopic rectal surgery.

Data from 8 studies^[19–21,25,27–29,31]^ involving 1710 patients (874 HT, 836 mLT) were meta-analyzed using a random-effects model. There was high heterogeneity among the studies (*I*^2^ = 90%, *P* < .00001). HT was associated with longer time to first flatus as mLT (WMD 0.29, 95% CI 0.11 to 0.48; *P* = .002; Fig. 11).

### 3.12. Rate of urinary dysfunction

**Figure 12.**
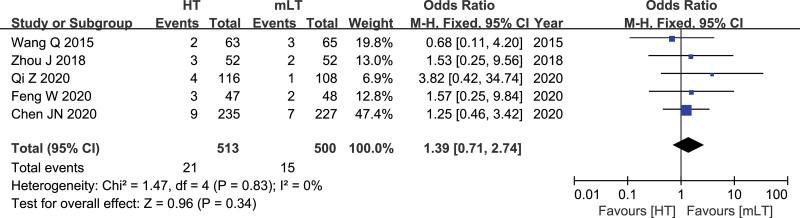
Forest plot of the rate of urinary dysfunction after HT or mLT laparoscopic rectal surgery.

Data from 5 studies^[17,25,28–30]^ involving 1013 patients (513 HT, 500 mLT) were meta-analyzed using a fixed-effects model. There was low heterogeneity among the studies (*I*^*2*^ = 0%, *P* = .83). HT was associated with similar rate of urinary dysfunction rate as mLT (OR 1.39, 95% CI 0.71 to 2.74; *P* = .34; Fig. 12).

### 3.13. Time of hospitalization

**Figure 13.**
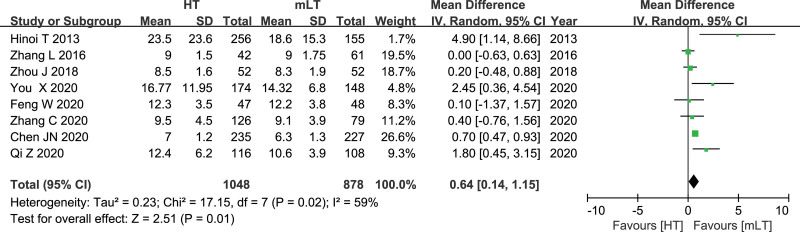
Forest plot of the time of hospitalization after HT or mLT laparoscopic rectal surgery.

Data from 8 studies^[16,20,25,27–31]^ involving 1926 patients (1048 HT, 878 mLT) were meta-analyzed using a random-effects model. There was moderate heterogeneity among the studies (*I*^2^ = 59%, *P* = .02). HT was associated with more hospitalization time as mLT (WMD 0.64, 95% CI 0.14 to 1.15; *P* = .01; Fig. 13).

### 3.14. Rate of recurrence and metastasis

**Figure 14.**
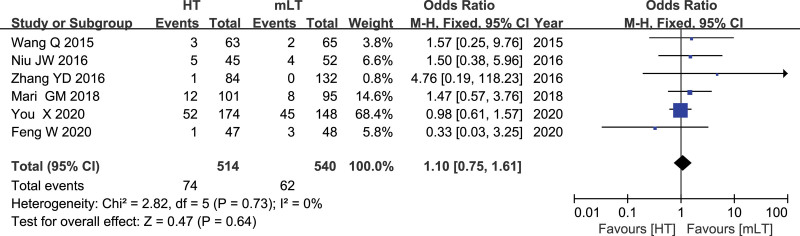
Forest plot of the rate of recurrence and metastasis after HT or mLT laparoscopic rectal surgery.

Data from 6 studies^[17,19,21,24,30,31]^ involving 1054 patients (514 HT, 540 mLT) were meta-analyzed using a fixed-effects model. There was low heterogeneity among the studies (*I*^2^ = 0%, *P* = .73). HT was associated with similar rate of recurrence and metastasis as mLT (OR 1.10, 95% CI 0.75 to 1.61; *P* = .64; Fig. 14).

### 3.15. 5-year Survival rate

**Figure 15.**
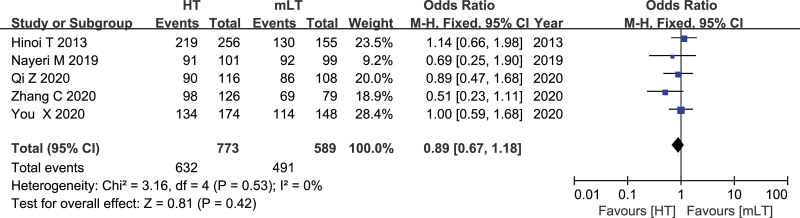
Forest plot of 5-year survival rate after HT or mLT laparoscopic rectal surgery.

Data from 5 studies^[16,24,27,29,31]^ involving 1362 patients (773 HT, 589 mLT) were meta-analyzed using a fixed-effects model. There was low heterogeneity among the studies (*I*^2^ = 0%, *P* = .53). HT was associated with a similar 5-year survival rate as mLT (OR 0.89, 95% CI 0.67 to 1.18; *P* = .42; Fig. 15).

### 3.16. Sensitivity analysis and publication bias

**Figure 16.**
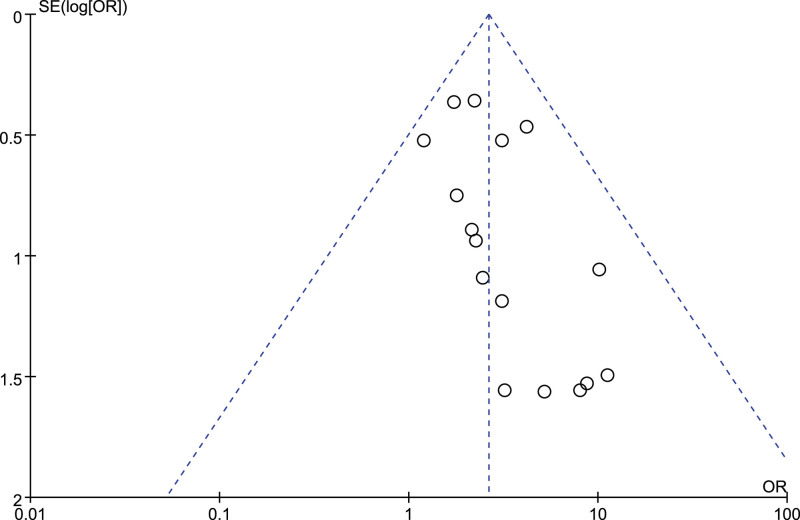
Funnel plot of studies reporting on anastomotic leakage.

For each of the meta-analyses described above, similar results were obtained after removing each study individually (data not shown). For each of the meta-analyses, risk of publication bias was assessed using funnel plots, which did not indicate severe publication bias; as an example, the funnel plot for the meta-analysis of anastomotic leakage is shown in Figure 16.

## 4. Discussion

This meta-analysis summarized the results of trials comparing preservation (mLT) and non-preservation (HT) of the left colic artery in laparoscopic rectal cancer surgery. HT and mLT approaches in laparoscopic rectal surgery were compared in terms of operating time, estimated blood loss, the number of harvested lymph nodes, the number of apical lymph node yield, the number of apical lymph node metastasis, rate of conversion to open surgery, rate of diverting stoma, rate of anastomotic leakage, time to first flatus, rate of urinary dysfunction, time of hospitalization, rate of recurrence and metastasis, 5-year survival rate. This meta-analysis demonstrated a statistically significant difference in operating time, rate of diverting stom, rate of anastomotic leakage, time to first flatus, rate of urinary dysfunction, time of hospitalization between the 2 surgical techniques. However, no significant differences were found in estimated blood loss, the number of harvested lymph nodes, the number of apical lymph node yield, the number of apical lymph node metastasis, rate of conversion to open surgery, rate of recurrence and metastasis, 5-year survival rate.

Anastomotic leakage is the most common serious postoperative complication after colorectal cancer surgery, and it is an important cause of mortality. Our meta-analysis of data from laparoscopic rectal cancer surgery patients suggests that HT is associated with significantly higher risk of anastomotic leakage than mLT. Of the many factors influencing anastomotic leakage, including blood supply, anastomotic tension, anastomotic technique, and nutritional status of patients, the 2 most important are blood supply and anastomotic tension.^[32,33]^ Preserving the left colic artery guarantees adequate blood supply to the colon proximal to the anastomosis,^[32,34]^ while removing this artery can significantly reduce perfusion of the proximal intestine.^[34,35]^ In addition to ensuring adequate blood supply, LT may ensure a sufficiently long free bowel to prevent a significant increase in anastomotic tension. This is consistent with the idea that LT can achieve tension-free anastomosis by cutting the descending branch of the left colic artery^[36]^ and contrasts with the suggestion that only HT can provide sufficiently long free bowel.^[32,33,37]^ It may be because the HT is more likely to affect the blood supply of the anastomotic, which leads to the unsatisfactory of the anastomotic, and therefore the prophylactic ileostomy is performed, which is also consistent with the results of our meta analysis. The results of our meta-analysis showed that the diverting stoma rate in the HT group was higher than that in the mLT group, which may be because the HT group may reduce the blood supply of the bowel, leading to anastomotic ischemia. The surgeons were not satisfied with the anastomosis, so they performed prophylactic ileostomy.

Another issue that needs our attention was that the consequence of variation of LCA. A recent systematic review reported that the pooled prevalence estimate of LCA absence was 1.2%, though the absence of a LCA is a rare occurrence, it may be associated with an important risk for anastomotic leak as a result of insufficient vascularization of the proximal colonic conduit.^[38]^ Five of the included studies mentioned the variation of LCA.^[18,19,25,26]^ Bifurcations of the IMA were divided into 4 patterns reported by Murono^[11]^: in type I, the LCA arose independently from the sigmoid artery (SA); in type II, the LCA and SA arose from the IMA at the same point; in type III, the LCA and SA had a common trunk; and in type IV, there was a defificit of the LCA. Huang’s study showed that type III IMA and lack of the Riolan were independent risk factors for anastomotic leakage.^[18]^ Surgeons should be aware that technical difficulties are likely to be more common in the type II and type III, as division of LCA at its origin may be more difficult due to its close proximity.

The available evidence indicates that mLT requires significantly longer operating time than HT, which may reflect the increased effort and skill needed to dissect lymph nodes and expose blood vessels. mLT may require greater surgical skills and longer operation time than HT.^[39]^ It is possible that operating time can decrease as surgeons gain experience: if surgeons are sufficiently experienced, mLT can lead to equally effective lymph node dissection as HT without excessive operating time,^[40]^ and this is supported by our meta-analysis. The extent of lymph node involvement in rectal cancer is the most important determinant of survival, so lymph node dissection is critical in radical resection of rectal tumors. Examination of an adequate number of nodes is critical for accurate staging and prediction of prognosis.^[41]^ Our meta-analysis showed no significant difference in the number of harvested lymph nodes, apical lymph node yield and apical lymph node metastasis between the HT and mLT procedures, both of which apparently can provide more than the minimum of 12 nodes needed for accurate tumor staging.^[42,43]^ One possible reason is that in most of the included studies, mLT was accompanied by lymph node dissection at the root of the inferior mesenteric artery. We believe that surgery preserving the left colic artery and dissecting lymph nodes at the root of this artery can satisfy the lymph node clearance required in laparoscopic rectal surgery.

In our meta-analysis the time to first flatus was slightly less in the mLT group than in the HT group. Sufficient blood supply and intact autonomic nerve function are important factors affecting postoperative gastrointestinal function recovery. The left paraaortic trunk runs along the left side of the aorta, passes through the posterior wall of the IMA and meets with the right trunk to form the superior inferior plexus. Thus, ligation of IMA at a level below LCA is less likely to injure the nerve trunk. High ligation of IMA can lead to decreased blood perfusion at the anastomotic, thus affecting the healing and functional recovery of the anastomotic.^[44,45]^ It is easy to understand that patients with earlier exhaust time and lower incidence of anastomotic leakage will have shorter hospital stay, which is consistent with the results of our meta analysis.

Urinary dysfunction in the rectal cancer surgery is usually dysuria by a damage of the hypogastric nerve plexus in the pelvis.^[46]^ Regardless of HT or mLT, the plane of ligation was above the pelvic cavity. Therefore, the results of our meta-analysis showed that there was no statistically significant difference in the incidence of urinary dysfunction between the 2 ligation methods. From the included studies, we found an interesting phenomenon, usually in rectal cancer patients after surgery, some patients will have sexual dysfunction, mostly caused by intraoperative pelvic autonomic nerve injury. Curiously, of the 16 studies we included, only 1 study^[31]^ described result on sexual dysfunction, whereas You’s study only described erectile dysfunction, so we could not combine data on sexual dysfunction. This may be because this topic is relatively private, and it is often difficult for researchers to get real data about it.

The available evidence also indicates similar recurrence and metastasis rate and 5-year survival rate after mLT or HT laparoscopic rectal surgery. The reason may be that all the studies included in our meta-analysis the 2 procedures allow dissection of a similar number of lymph nodes, apical lymph node yield and apical lymph node metastasis. The number of lymph nodes dissected is an important factor for the prognosis of patients.

The quality of studies in our review was relatively high. Nevertheless, the results of our meta-analysis should be interpreted with caution given several limitations. First, heterogeneity was detected in meta-analyses of 2 variables (operating time, estimated blood loss, the number of harvested lymph nodes, the number of apical lymph node yield, time to first flatus and time of hospitalization), which may reduce the reliability of these analyses, although we did compensate by using a random-effects model. Second, some meta-analyses involved fewer patients than others, which may also affect their reliability. Third, transforming skewed distributions (media/range) to normal distributions (mean/SD) may introduce bias. Fourth, although an extensive literature search was done, we may have missed some unpublished studies.

## 5. Conclusion

In summary, the available evidence suggests that preserving the left colic artery can decrease the incidence of anastomotic leakage and rate of diverting stoma, time to first flatus and hospitalization was shorter, without affecting intraoperative blood loss, number of harvested lymph nodes, apical lymph node yield, apical lymph node metastasis, rate of conversion to open surgery, rate of urinary dysfunction was lower, recurrence and metastasis rate and 5-year survival rate. Therefore, we believe that preserving the left colic artery is a safe, effective technique for patients with rectal cancer. At the same time, large RCTs are needed to confirm and extend these findings.

## Author contributions

**Data curation:** Chuanyuan Liu, Lei Zhang.

**Funding acquisition:** Wu Zhong.

**Investigation:** Junqiao Zhong, Xianping He.

**Methodology:** Chuanyuan Liu, Junqiao Zhong.

**Resources:** Chuanfa Fang.

**Validation:** Laiyang Xia.

**Visualization:** Hongquan Liu.

**Writing – original draft:** Wu Zhong.

**Writing – review & editing:** Wu Zhong, Zhengyun Zuo, Leichang Zhang.
